# Multivariable analysis for predicting lower limb muscular strength with a hip-joint exoskeleton

**DOI:** 10.3389/fbioe.2024.1431015

**Published:** 2024-10-24

**Authors:** Byungmun Kang, Changmin Lee, Dongwoo Kim, Hwang-Jae Lee, Dokwan Lee, Hyung Gyu Jeon, Yoonmyung Kim, DaeEun Kim

**Affiliations:** ^1^ Biological Cybernetics Lab, School of Electrical and Electronic Engineering, Yonsei University, Seoul, Republic of Korea; ^2^ Research Institute of Future City and Society, Yonsei University, Seoul, Republic of Korea; ^3^ Bot Fit T/F, New Biz T/F, Samsung Electronics, Suwon, Republic of Korea; ^4^ Sports Medicine and Athletic Training Lab, Department of Physical Education, Yonsei University, Seoul, Republic of Korea; ^5^ University College, Yonsei University International Campus, Incheon, Republic of Korea

**Keywords:** Bot Fit, hip-joint exoskeleton, muscular strength, multivariable analysis, resistance exercise

## Abstract

**Introduction:**

Advancements in exercise science have highlighted the importance of accurate muscular strength assessments for optimizing performance and preventing injuries.

**Methods:**

We propose a novel approach to measuring muscular strength in young, healthy individuals using Bot Fit, a hip-joint exoskeleton, during resistance exercises. In this study, we introduced performance metrics to evaluate exercise performance during both short and extended durations of three resistance exercises: squats, knee-ups, and reverse lunges. These metrics, derived from the robot’s motor signals and sEMG data, include initial exercise speed, the number of repetitions, and muscle engagement. We compared these metrics against baseline muscular strength, measured using standard fitness equipment such as one-repetition maximum (1RM) and isometric contraction tests, conducted with 30 participants aged 23 to 30 years.

**Results:**

Our results revealed that initial exercise speed and the number of repetitions were significant predictors of baseline muscular strength. Using statistical multivariable analysis, we developed a highly accurate model ( R=0.884 , adj. $R^{2}=0.753$ , *p*-value <0.001 ) and an efficient model (with all models achieving R>0.87 ) with strong explanatory power.

**Conclusion:**

This model, focusing on a single exercise (squat) and a key performance metric (initial speed), accurately represents the muscular strength of Bot Fit users across all three exercises. This study expands the application of hip-joint exoskeleton robots, enabling efficient estimation of lower limb muscle strength through resistance exercises with Bot Fit.

## 1 Introduction

In recent years, progress in medical technology, digital healthcare, and environmental innovations has significantly enhanced overall wellbeing. These advances have resulted in tools that not only monitor but also support health. Among these, exercise is essential for both physical and mental wellness, serving a crucial role in preventing and managing chronic diseases (Rippe and Hess, 1998).

To fully benefit from exercise, accurate assessment of an individual’s physical capabilities, particularly muscular strength, is essential. Muscular strength is a key indicator of overall fitness, influencing daily functional abilities and athletic performance (Garber et al., 2011). Accurate strength assessments enable personalized exercise programs that maximize results while minimizing injury risk, promoting optimal health outcomes (Scott et al., 2019).

Traditional methods for assessing muscular strength, such as the One-Repetition Maximum (1RM) test, external weight resistance exercises (Verdijk et al., 2009), handheld dynamometry (O’Shea et al., 2007), isokinetic dynamometers (Baltzopoulos, 2007), and isometric strength tests (Essendrop et al., 2001), have been used for decades. While effective for determining strength, these methods often require specialized equipment and controlled environments, limiting accessibility outside clinical or gym settings. Additionally, they typically provide static measurements of strength, lacking real-time feedback during dynamic exercises (Baltzopoulos, 2007; Essendrop et al., 2001).

Recent technological advancements have introduced wearable robots, such as exoskeletons, to address these limitations. Initially developed for rehabilitation and mobility assistance, exoskeletons have evolved to support resistance exercises, offering a portable and versatile means of assessing and improving muscular strength (Cornwall, 2015; Azimi et al., 2018). These devices can provide real-time feedback, which is crucial for optimizing exercise performance and ensuring safety. For instance, a sparse Gaussian process (SGP) has been used to create a probabilistic model of knee movement, enhancing stability by predicting the relationship between knee and hip movements and setting boundary limits Chen et al. (2023). Additionally, electromyography (EMG) signals have been used to evaluate active movements, estimate joint torque, and propose practical robotic motion control to improve exoskeleton-based rehabilitation Gui et al. (2019). Central pattern generators (CPGs) have also been utilized to adjust users’ gait trajectories in real time, ensuring proper alignment between the exoskeleton and the user’s gait Kou et al. (2024). Over the past decade, there has been significant progress in lower-limb rehabilitation exoskeleton research Wen et al. (2024); Shi et al. (2019).

One such innovation is the Bot Fit exoskeleton, developed by Lee et al. (Lee et al., 2023; Kim et al., 2018). This device provides both resistance and assistance during exercises, making it suitable for a wide range of users, including older adults and those undergoing rehabilitation. Bot Fit enhances physical performance by offering resistance, aiding in movement, and delivering real-time feedback, such as voice guidance and posture alerts via a smartphone, thereby improving exercise adherence.

With the growing use of wearable technologies, there is an increasing demand for accurate and reliable metrics to estimate muscular strength during resistance exercises. While traditional strength assessments are valuable, they are often insufficient in dynamic, real-time contexts. Surface electromyography (sEMG) has proven effective for measuring muscle activity during exercise (De Luca, 2002; Merletti and Farina, 2016). However, sEMG alone cannot quantify muscular strength due to the nonlinearity between muscle strength and sEMG signals Liu et al. (2022). Musculoskeletal models Zhang et al. (2016) are often used to estimate strength from sEMG data, but these models are complex and require careful consideration of variables such as muscle length and contraction velocity (Buchanan et al., 2004; Sartori et al., 2012).

Although some studies, such as those by Staudenmann et al. (2010), have developed methods for estimating force from sEMG signals, these approaches face challenges related to accuracy and generalizability. Furthermore, many existing studies focus on specific populations or exercise protocols Mokri et al. (2022), limiting their broader applicability. This paper emphasizes the need for more advanced methods to accurately estimate muscular strength across diverse users and exercise types, particularly with the integration of wearable technologies like exoskeleton robots.

In this paper, we propose a novel method for estimating lower limb muscular strength during three different resistance exercises using the Bot Fit exoskeleton. We conducted an experimental study with thirty healthy participants, introducing performance metrics based on motor signals from the device and sEMG signals from the users’ movements, without relying on complex musculoskeletal models. Through multivariable analysis, we investigated the most effective metrics for representing muscle strength. Based on these findings, we present a model for estimating users’ muscular strength through exercise performance while wearing the Bot Fit exoskeleton.

## 2 Methods

### 2.1 Experimental platform

#### 2.1.1 Hip-joint exoskeleton & wireless sEMG sensor

**FIGURE 1:**
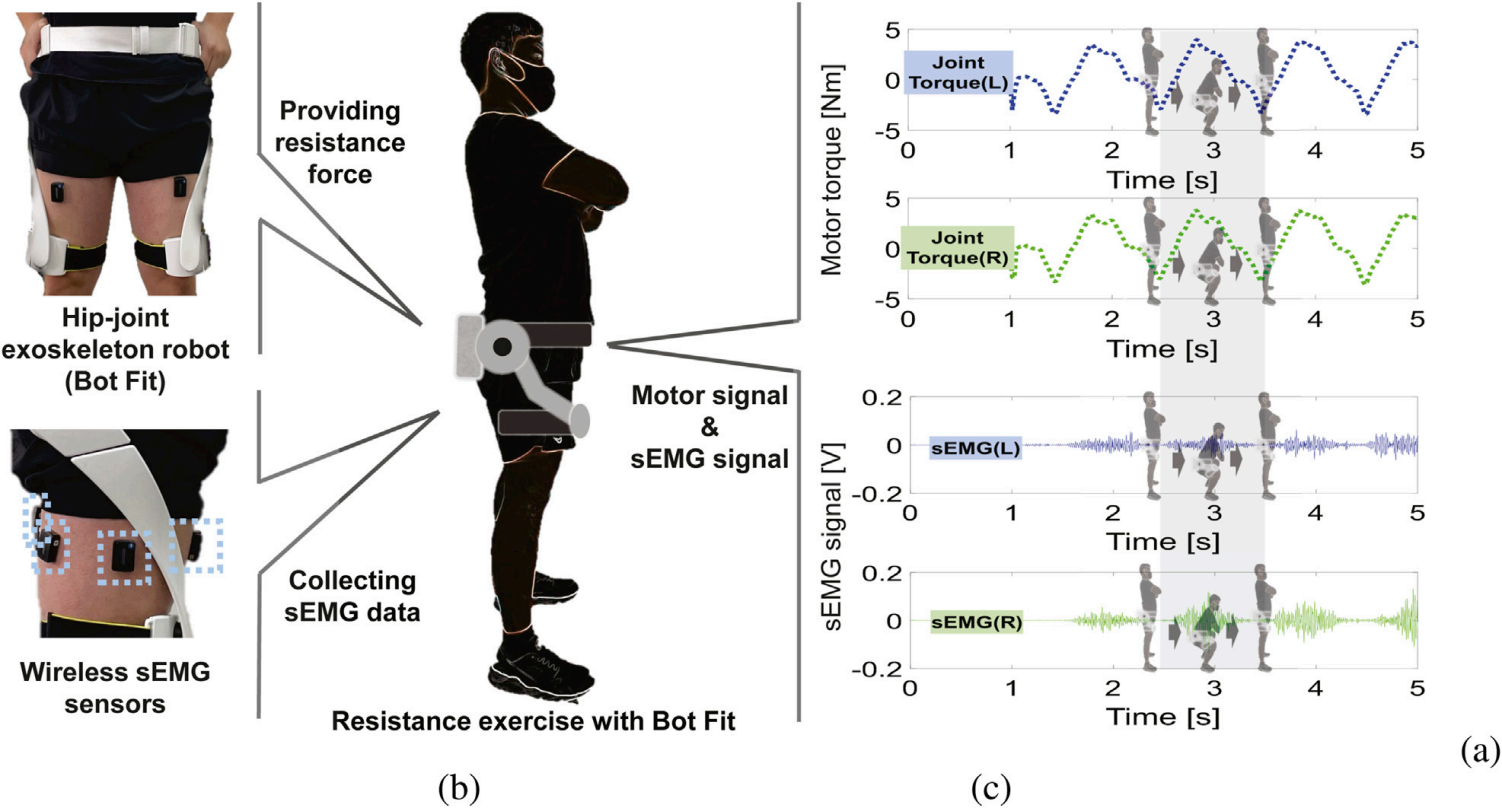
Hip-joint Exoskeleton Robot (Bot Fit) for Resistance Exercise. **(A)** Images of the exoskeleton robot used to provide adaptive hip-joint resistance for participants, along with the wireless sEMG sensors used to measure muscle activity during the exercises. **(B)** Schematic illustration of a participant equipped with the exoskeleton robot and wireless sEMG sensors. **(C)** Joint torque (motor signal) and sEMG signals obtained during a squat exercise.

As shown in Figures 1A, B, the Bot Fit, a hip-joint exoskeleton developed by Samsung Electronics Co., Ltd. (Korea), applies resistance torque to the hip joints during exercise. This lightweight (2.9 kg), slim, and comfortable device includes actuators for each hip joint, an adjustable fabric waist belt, and thigh frames to transmit resistance torque. Equipped with BLDC (Brushless Direct Current) motors near the hip joints, it generates torque (Figure 1C) tailored to the exercise type, with sensors measuring movement direction and angle. An IMU sensor on the back monitors activity and adjusts torque based on speed and rhythm. Bot Fit offers five resistance levels (1R–5R) and can generate torque up to 10 Nm using its two high-speed BLDC motors (Kim et al., 2018; Lee et al., 2023). Additionally, a time-delayed self-feedback controller (DOFC) algorithm ensures safe and adaptive resistance exercises tailored to the user’s movements (Lim et al., 2019).

We used wireless sEMG sensors (Delsys Trigno System, Boston, MA, United States) to measure thigh muscle activity at a sampling rate of 2000 samples/s (Figures 1A, C). These sensors were attached to four regions on each thigh: the rectus femoris, vastus lateralis, biceps femoris, and semitendinosus muscles. The sensors were placed at the midpoint of the targeted muscles, aligned parallel to the muscle fibers. To ensure accurate sEMG signal acquisition, the skin was prepared by removing oil and sweat, with hair removal performed if necessary, following standard guidelines (Hermens et al., 1999).

### 2.2 Participants & experimental protocol

#### 2.2.1 Participants

This study included 30 healthy adults (19 males, 11 females) aged 23 to 30, recruited through promotional activities at a fitness center operated by the research team. Participants were informed about the study, provided written consent, and completed ethics training in accordance with the Institutional Review Board (IRB) protocol at Yonsei University (Registration number: 7001988-202305-HR-1538-04).

Participants were selected based on the following criteria: 1) They were young adults without significant medical conditions, such as cardiovascular, musculoskeletal, or neurological disorders. 2) They regularly engaged in light physical activity, such as jogging, yoga, or light weightlifting, approximately 1–2 times per week. 3) Their health was assessed through body mass index (BMI) and blood pressure measurements on Day 1 of pre-measurement, ensuring all health metrics fell within a healthy range. 4) All participants were capable of daily movement and walking without the need for mobility aids or assistance.

Exclusion criteria were as follows: 1) Participants who were uncomfortable wearing the robotic exoskeleton or attaching wireless sEMG sensors for muscle activity monitoring were excluded. 2) Individuals with severe communication impairments, major medical conditions (e.g., heart disease or lower limb disorders), or a BMI of 30 kg/^2^ or higher (the normal range is 18.5–24.9 kg/^2^ (Consultation, 2000)) were excluded due to safety concerns related to the exoskeleton robot. 3) Additionally, participants could be excluded at the researcher’s discretion if deemed unsuitable for the study.

Overall, the study group consisted of healthy individuals who engaged in light to moderate exercise and maintained relatively healthy lifestyles. Specific details about the participants’ demographics and health metrics are provided in Table 1.

**TABLE 1:** Characteristics of the participants. SD standard Deviation.

Characteristic	Values
Sex (male/female)	19/11
Age (mean ± SD)	26.1 ± 2.8 [years]
Height (mean ± SD)	170.93 ± 8.5 [cm]
Weight (mean ± SD)	66.43 ± 9.8 [kg]
Resting HR (mean ± SD)	81 ± 9.43 [BPM]
Blood pressure High (mean ± SD)	109.4 ± 9.99 [mmHg]
Blood pressure Low (mean ± SD)	70.67 ± 7.78 [mmHg]
ASM (mean ± SD)	28.5 ± 5.91 [kg]
BMI (mean ± SD)	22.6 ± 2.31 [kg/ $m^{2}$ ]

ASM, means appendicular skeletal muscle mass and BMI, means body mass index.

#### 2.2.2 Experimental protocols: Pre-measurement phase

**FIGURE 2:**
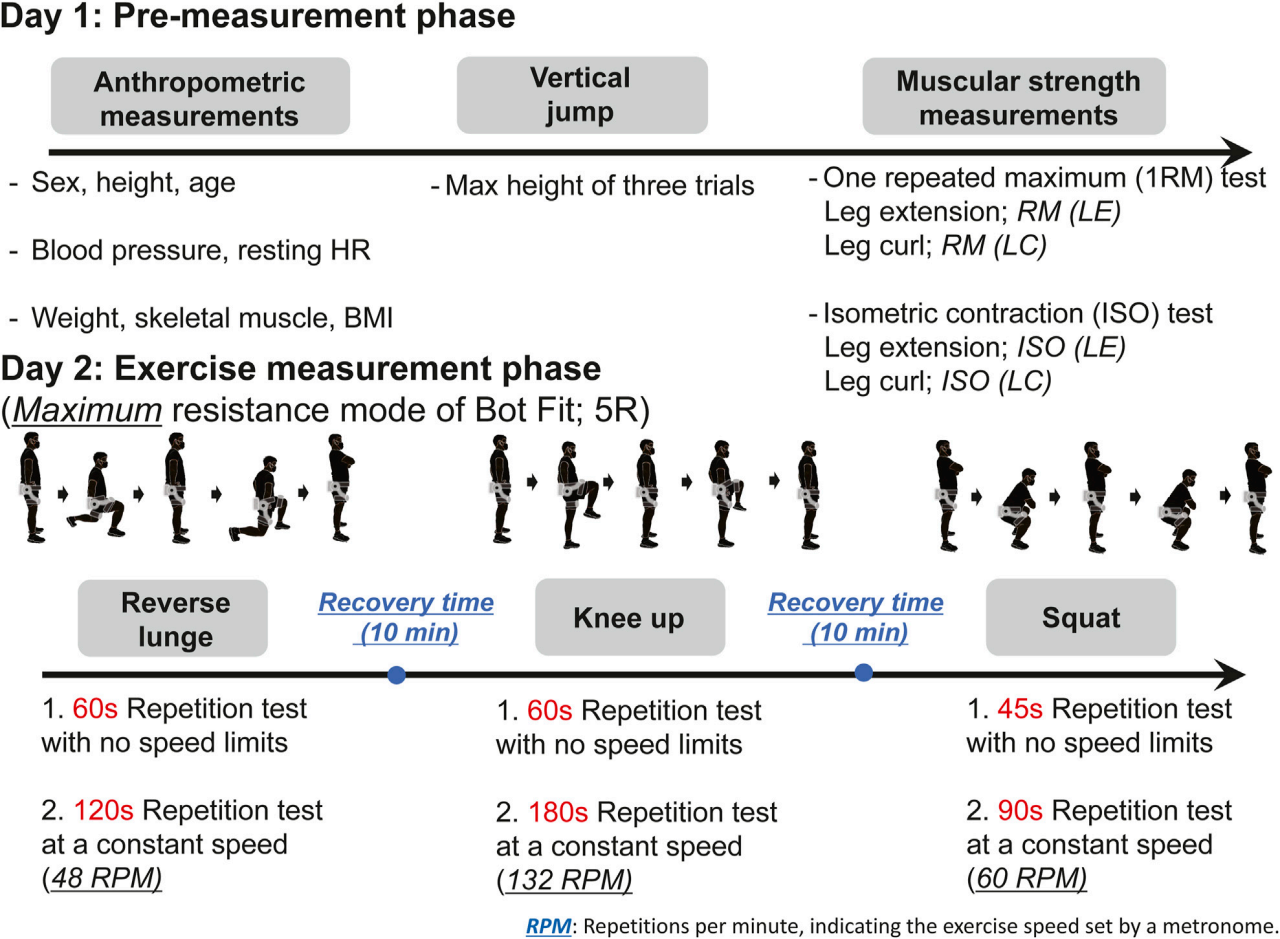
Experimental protocol. During the pre-measurement phase, participants underwent assessments including a vertical jump test, a 1RM test using a leg curl and extension fitness machine, and an isometric contraction test focusing on leg curls (LCs) and leg extensions (LEs) using a hand-held dynamometer. These assessments were used to derive muscle parameters indicative of baseline muscular strength. During the exercise measurement phase, participants performed three resistance exercises while wearing the exoskeleton robot, and sEMG signals were recorded simultaneously. Each resistance exercise was performed under two different protocols: one with short bursts of repetitions at an uncontrolled speed, and another with a relatively long duration at a fixed speed. Adequate rest periods were provided between exercises.

The experimental protocol consisted of two phases of measurement for each participant: a pre-measurement phase and an exercise measurement phase using the maximum resistance mode of Bot Fit (Figure 2).

In the pre-measurement phase, baseline muscular strength was evaluated using standard fitness equipment without Bot Fit. Muscle parameters reflecting each participant’s baseline strength were collected (Table 2). Muscular power was assessed through a vertical jump (VJ) test, where the highest score from three trials was recorded. This test involved participants bending their knees and jumping vertically (Wisløff et al., 2004; Iossifidou et al., 2005).

**TABLE 2:** Description of muscle parameters.

Parameters	Description
Total performance	Comprehensive muscular strength index; calculated as the sum of z-scores for VJ, RM (LE + LC, Weight), ISO (LE + LC, Weight) (*p*-value: 0.125)
VJ	The Maximum Height of three trials (*p*-value: 0.923)
RM (LE + LC, Weight)	Normalization values of sum of the measured values by leg extension (LE) and leg curl (LC) with fitness machine to the participant’s weight (*p*-value: 0.079)
RM (LE, Weight)	Normalization of the measured value of leg extension (LE) with fitness machine to the participant’s weight (*p*-value: 0.14)
RM (LC, Weight)	Normalization of the measured value of leg curl (LC) with fitness machine to the participant’s weight (*p*-value: 0.216)
ISO (LE + LC, Weight)	Normalization values of sum of the measured values by leg extension (LE) and leg curl (LC) with isometric contraction to the participant’s weight (*p*-value: 0.552)
ISO (LE, Weight)	Normalization of the measured value of leg extension (LE) with isometric contraction to the participant’s weight (*p*-value: 0.902)
ISO (LC, Weight)	Normalization of the measured value of leg curl (LC) with isometric contraction to the participant’s weight (*p*-value: 0.072)

*p*-value denotes the significance probability of normality test (Shapiro-Wilk test).

Muscular strength, assessed via 1RM, was measured using leg extension (LE) and leg curl (LC) machines (Life Fitness, Franklin Park, Illinois, United States). In the LE test, participants, while seated, lifted the maximum weight by extending the knee and foot, targeting the rectus femoris muscle (Krisnan et al., 2014). In the LC test, participants lay prone and flexed the knee and foot to lift the maximum weight, engaging the biceps femoris (Llurda-Almuzara et al., 2021).

Lower limb muscle strength was also measured using a hand-held dynamometer (EasyForce, GMT Ltd, Bury Saint Edmunds, United Kingdom). The dynamometer was secured to the ankle while participants, seated with knees at a 90-degree angle, exerted force for 5 s to measure the strength of the rectus femoris and biceps femoris muscles (Gaudet and Handrigan, 2020; Sinacore et al., 2017).

Before the pre-measurement exercises, participants completed a warm-up led by a researcher specializing in exercise physiology. For explosive strength exercises such as the vertical jump and 1RM test, participants used equipment like stretching bands to activate lower body muscles through contraction and relaxation (Herrera and Osorio-Fuentealba, 2024). A brief massage was also provided prior to the experiment. For each exercise, participants performed preliminary practice, including five practice jumps before the vertical jump measurement, and five repetitions at 30% of their body weight before the 1RM test and isometric exercises. The same warm-up and stretching routine was followed before the exercise measurement phase on Day 2.

The pre-measurement phase took place on Day 1 of the experiment. To avoid influencing the subsequent exercise measurements, the exercise measurement phase was conducted 1–2 days later. This rest period was chosen because the exercises on Day 1 were not of high intensity, and short rest intervals in low-load resistance training typically do not interfere with muscle recovery, ensuring performance in the following session remained unaffected (Fink et al., 2017).

#### 2.2.3 Experimental protocols: Exercise measurement phase

On Day 2, during the exercise measurement phase, participants performed three exercises using Bot Fit, as shown in Figure 2. These exercises were chosen for their focus on movements involving the pelvis and hip joints, which are compatible with Bot Fit’s design. The exercises involved bilateral leg movements in the sagittal plane, emphasizing flexion and extension without abduction, and aligning with Bot Fit’s resistance application to prevent lateral pelvic motion.

The muscles activated during these exercises were monitored using sEMG signals, including the rectus femoris, vastus lateralis, biceps femoris, and semitendinosus (Slater and Hart, 2017; Muyor et al., 2020). These muscles were selected due to their critical involvement in Bot Fit exercises (Cabral et al., 2023; Lee et al., 2022). Additionally, the Day 1 pre-measurement tests, such as the Vertical Jump and 1RM tests, also target the quadriceps (Krisnan et al., 2014) and posterior thigh muscles (Llurda-Almuzara et al., 2021), which are the same muscles engaged during the Day 2 exercises (Lee et al., 2022; Narici et al., 1989). This similarity in muscle involvement was a key factor in selecting these exercises for accurate strength assessment.

During the measurement phase, participants performed resistance exercises under two distinct experimental conditions aimed at assessing different physical performance aspects. In the first condition, participants executed the exercises at maximum speed without a time limit for short durations (60 or 45 s). This condition, mimicking n-RM without speed constraints, allowed for the measurement of peak strength and maximum effort under high-intensity, short-duration circumstances (Willardson and Bressel, 2004).

**FIGURE 3:**
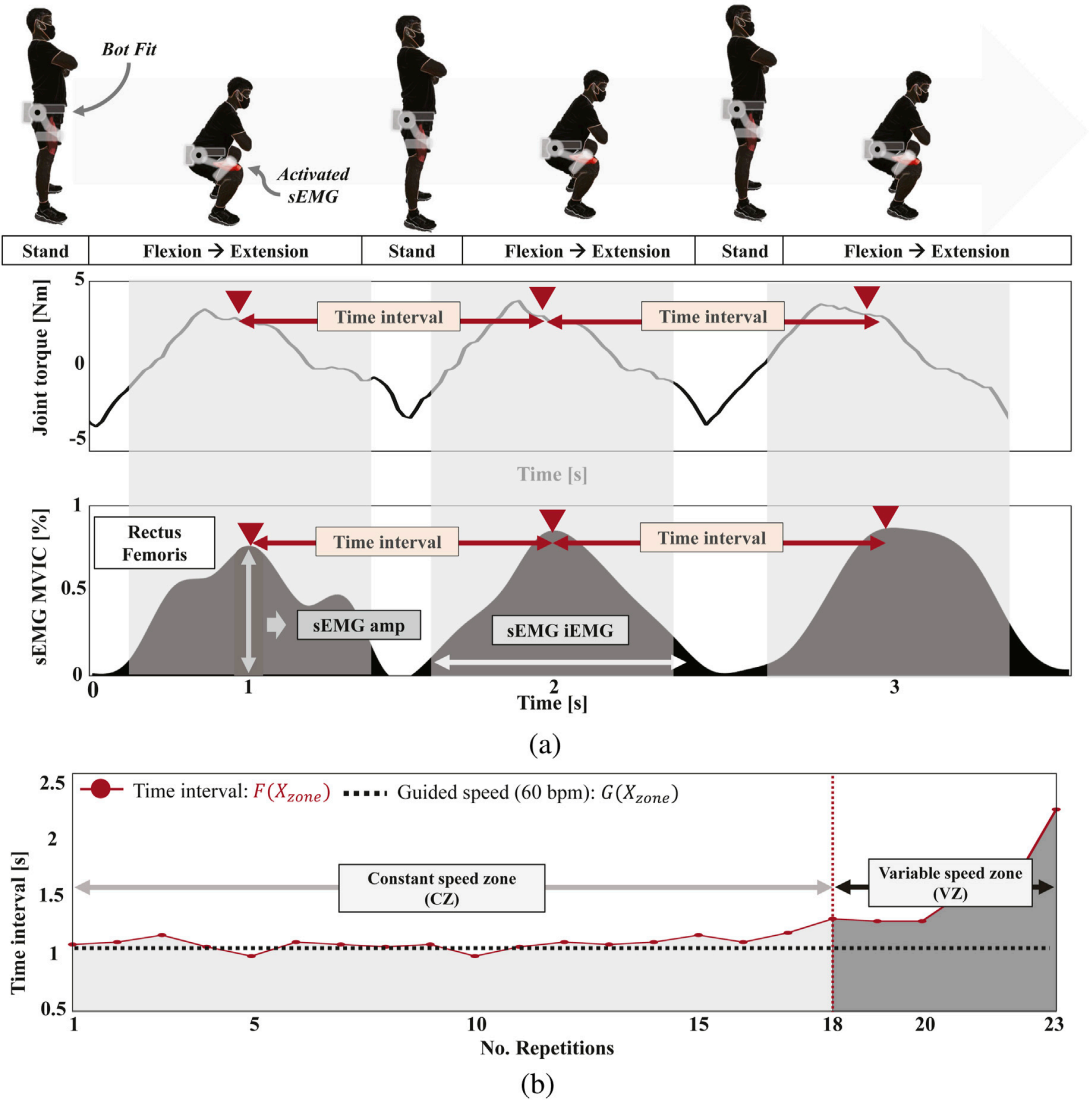
Examples of sEMG and Bot Fit motor signal processing during a 90-s repetition test at a constant speed (60 RPM) for squats. **(A)** Joint torque from Bot Fit and the raw sEMG signal recorded from lower limb muscles during squats in maximum resistance mode. The time interval between peak values of each signal represents the speed of exercise performance per repetition. **(B)** A graph of the time intervals from **(A)**, where segments maintaining exercise speed are designated as the constant speed zone, and those unable to maintain speed are marked as the variable speed zone. This analysis evaluates how effectively each participant uses their muscular strength to maintain speed.

The second condition, known as the “constant speed” condition, required participants to maintain a fixed speed for longer durations (180, 120, or 90 s), with a metronome ensuring consistency. We defined this speed as RPM (Repetitions per minute). This condition focused on muscular endurance by identifying the point of muscle fatigue, which occurred when participants could no longer sustain the designated pace during exercises like squats (Figure 3). By maintaining a constant speed for 30–45 s, this setup emphasized endurance over pure strength, promoting sustained muscular effort. The controlled speed also minimized variability, allowing for a more objective assessment of endurance, fatigue, and efficiency. Previous studies emphasize the importance of repetition speed in strength development, with faster speeds leading to greater strength gains (Westcott et al., 2001). Given the connection between strength and endurance, this method provides an indirect estimation of endurance through strength performance (Stone et al., 2006; Vaara et al., 2012).

These two experimental conditions offer complementary insights: the maximum speed condition assesses peak strength and short-term performance under high-intensity conditions, while the constant speed condition evaluates endurance, fatigue management, and sustained effort over time. This combined approach allows for a more comprehensive analysis of physical performance, capturing both the limits of strength and the ability to sustain exercise over longer periods, providing a well-rounded evaluation of the Bot Fit resistance protocol (Lander et al., 2009).

All participants followed a specific exercise sequence consisting of reverse lunges, knee-ups, and squats (Figure 2). First, a repetition test lasting either 60 or 45 s without speed limits was conducted, followed by a constant-speed repetition test lasting either 180, 120, or 90 s for each exercise. This approach ensured reliable measurement and consistency throughout the experiment. The constant speed was determined by the duration of the test, with speeds tailored to each exercise: 48 RPM for reverse lunges, 132 RPM for knee-ups, and 60 RPM for squats. These values were chosen to reflect the level of difficulty and muscle engagement required for each movement.

The main challenges in our exercise protocol were maintaining proper posture, correct speed, and pre-exercise stability. Bot Fit provided real-time alerts, and researchers monitored posture. In the knee-up exercise at 132 RPM, participants maintained a knee angle below 65°, which was also applied to the lunge and squat for consistent control.

Resting heart rates were checked before each exercise in Table 1, and if elevated above 100 BPM of heart rate, the experiment was paused until the heart rate returned to normal to ensure stable conditions. In addition, all protocols were performed 2 hours after meals, a design choice made to account for the potential effects of food intake on exercise performance (Farah and Gill, 2013).

### 2.3 Data acquisition and processing

#### 2.3.1 Data acquisition

Comprehensive data were collected, including muscle activity, vertical jump performance, 1RM test results, isometric contraction data, and the number of repetitions for all exercises performed with Bot Fit. Wireless sEMG sensors captured muscle signals during each exercise, and Bot Fit’s motor data were used to validate the accuracy of the sEMG signals. The alignment between the repetitions recorded by researchers and those detected by the sEMG sensors confirmed their synchronization, reinforcing the reliability of the motor signals and the study’s conclusions.

Additionally, comparing the two signals confirmed that Bot Fit’s motor data accurately reflected the muscle movements captured by the sEMG sensors, further ensuring the reliability of the motor signals.

#### 2.3.2 Bot Fit motor signal processing

For Bot Fit’s motor signal processing, Figure 3A shows the joint torque measured from Bot Fit’s motor during a squat exercise at 60 RPM. The torque peaks during the flexion and extension phases of the squat. We measure the time intervals between these peaks, as shown in Figure 3B. The interval where the 60 RPM speed is maintained is defined as the “constant speed zone,” while intervals where the speed deviates are labeled the “variable speed zone.” The same method was applied to determine time intervals for reverse lunges and knee-ups using Bot Fit’s motor signals.

#### 2.3.3 sEMG signal processing

All sEMG signals underwent thorough preprocessing in each measurement phase, including the application of a notch filter to eliminate power line interference, a high-pass filter to remove mechanical noise, and a band-pass filter (20 Hz–500 Hz) to retain the relevant sEMG signal frequencies.

In the pre-measurement phase, peak muscle activation signals from the 1RM and isometric contraction tests were used to normalize the sEMG signals. This normalization, based on maximum voluntary isometric contraction (MVIC), enables objective comparisons by expressing EMG signal intensity as a percentage of the highest RMS value from the MVIC (Soderberg, 1992; Winter 2009; Lawrence and De Luca, 1983). The MVIC of the sEMG signal $\left({MVIC}_{ex}\right)$ is calculated using the formula:

$${\text{MVIC}}_{ex}=\frac{{\text{sEMG}}_{ex}-{\text{sEMG}}_{rest}}{{\text{sEMG}}_{max}-{\text{sEMG}}_{rest}}$$ (1)

In Equation 1, ${\text{sEMG}}_{max}$ represents the maximum sEMG signal obtained during the 1RM and isometric contraction tests. ${\text{sEMG}}_{ex}$ denotes the sEMG signals recorded during resistance exercises such as reverse lunges, knee-ups, and squats, as shown in the MVIC of Figure 3B, while ${\text{sEMG}}_{rest}$ denotes the resting sEMG signals.

### 2.4 Evaluation metrics for multivariable analysis

#### 2.4.1 Dependent variables: Muscle parameters

Our model focuses on a muscle parameter that serves as an indicator of participants’ baseline muscular strength. This parameter includes metrics such as VJ, RM (LE + LC, weight), ISO(LE + LC, weight), and total performance. Measured in the pre-measurement phase and detailed in Table 2, these metrics provide insights into lower-limb muscular power (VJ) and strength relative to body weight (RM and ISO) and act as an overall measure of baseline muscular strength (total performance). Weight normalization for RM (LE + LC, weight) and ISO (LE + LC, weight) ensures an unbiased comparison among participants Bohannon (2009). The total performance is a composite indicator calculated by summing the z scores of VJ, RM (LE + LC, weight), and ISO (LE + LC, weight) Legarra-Gorgoñon et al. (2023).

#### 2.4.2 Independent variables: Performance metrics

We developed performance metrics as independent variables for the regression model. These metrics were derived from motor and sEMG signals measured during three resistance exercises (squats, knee-ups, and lunges) performed while wearing Bot Fit under two experimental conditions (detailed in Table 3).

**TABLE 3:** Proposed sEMG signal analysis-based performance metrics with Bot Fit at exercise measurement phase.

Metrics	Explanation & Remarks
Number of repetition (NR)	Number of repetitions of exercise
	Squat NR Max: NR of squat with no speed limits in 45 s [*p*-value: 0.22] Kneeup NR Max: NR of knee-up with no speed limits in 60 s [*p*-value: 0.67] Lunge NR Max: NR of reverse lunge with no speed limits in 60 s [*p*-value: 0.602]
	Squat NR Const: NR of squat with 60 RPM in 90 s [*p*-value: 0.32] Kneeup NR Const: NR of knee-up with 132 RPM in 180 s [ < 0.001] Lunge NR Const: NR of reverse lunge with 48 RPM in 120 s [*p*-value: 0.065]
Initial speed (IS)	Average exercise speed during the initial repetitions of the exercise
	Squat IS Max: IS of squat with no speed limits for 10 repetitions [*p*-value: 0.211] Kneeup IS Max: IS of knee-up with no speed limits for 30 repetitions [*p*-value: 0.34] Lunge IS Max: IS of reverse lunge with no speed limits for 10 repetitions [*p*-value: 0.13]
	Squat IS Const: IS of squat with 60 RPM for 10 repetitions [*p*-value: 0.004] Kneeup IS Const: IS of knee-up with 132 RPM for 30 repetitions [ < 0.001] Lunge IS Const: IS of reverse lunge with 48 RPM for 10 repetitions [*p*-value: 0.53
Constant speed zone (CZ)	Number of exercise repetitions during the zone that adheres the constant speed (Figure 3B)
	Squat CZ: CZ of squat with 60 RPM in 90 s [*p*-value: 0.001] Kneeup CZ: CZ of knee-up with 132 RPM in 180 s [*p*-value: 0.032] Lunge CZ: CZ of reverse lunge with 48 RPM in 180 s [*p*-value: 0.085]
sEMG amp	Difference in amplitude of the sEMG signal between the initial 10 s and the final 10 s (Figure 3A)
	Squat sEMG amp Max: sEMG amp of squat with no speed limits in 45 s [ < 0.001] Kneeup sEMG amp Max: sEMG amp of knee-up with no speed limits in 60 s [*p*-value: 0.012] Lunge sEMG amp Max: sEMG amp of reverse lunge with no speed limits in 60 s [ < 0.001]
	Squat sEMG amp Const: sEMG amp of squat with 60 RPM in 60 s [*p*-value: 0.031] Kneeup sEMG amp Const: sEMG amp of knee-up with 132 RPM in 180 s [*p*-value: 0.033] Lunge sEMG amp Const: sEMG amp of reverse lunge with 48 RPM in 180 s [*p*-value: 0.627]
sEMG iEMG	Difference in iEMG of the sEMG signal between the initial 10 s and the final 10 s (Figure 3A)
	Squat sEMG iEMG Max: sEMG iEMG of squat with no speed limits in 45 s [*p*-value: 0.01] Kneeup sEMG iEMG Max: sEMG iEMG of knee-up with no speed limits in 60 s [ < 0.001] Lunge sEMG iEMG Max: sEMG iEMG of reverse lunge with no speed limits in 60 s [*p*-value: 0.001]
	Squat sEMG iEMG Const: sEMG iEMG of squat with 60 RPM in 60 s [*p*-value: 0.774] Kneeup sEMG iEMG Const: sEMG iEMG of knee-up with 132 RPM in 180 s [*p*-value: 0.403] Lunge sEMG iEMG Const: sEMG iEMG of reverse lunge with 48 RPM in 180 s [*p*-value: 0.627]

*p*-value denotes the significance probability of normality test (Shapiro-Wilk test).

The first metric, Number of Repetitions (NR), measures strength by counting the total repetitions performed. It is represented as Squat NR Max, Kneeup NR Max, and Lunge NR Max for the first experimental condition, and Squat NR Const, Kneeup NR Const, and Lunge NR Const for the second condition.

The second metric, Initial Speed (IS), assesses participants’ ability to sustain the initial speed guided by a metronome. It is calculated by averaging the speed of the initial 10 repetitions for squats or 30 repetitions for knee-ups and lunges. This metric is also categorized as Squat IS Max, Kneeup IS Max, and Lunge IS Max for the first condition, and Squat IS Const, Kneeup IS Const, and Lunge IS Const for the second.

The third metric, Constant Speed Zone (CZ), evaluates the ability to maintain a constant speed during repetitions until fatigue. CZ is calculated at the 18-s mark based on a threshold of speed change (Figure 3B) and is labeled as Squat CZ, Kneeup CZ, and Lunge CZ.

These three metrics are derived from Bot Fit’s joint torque, capturing the user’s movement, and can also be extracted using peak sEMG values. Unlike previous studies using machine learning to predict movement from sEMG signals Mokri et al. (2022); Kyeong et al. (2022), our approach relies on peak values directly tied to user movement (Figure 3A). Since motor and sEMG signals are synchronized, we used Bot Fit’s motor signals for these metrics in the analysis.

The fourth metric, sEMG Amplitude, measures the change in sEMG signal amplitude between the initial and final 10 s of exercise. This reflects muscle activation intensity and contraction strength (Ryu et al., 2016). It is represented as Squat sEMG amp Max, Kneeup sEMG amp Max, and Lunge sEMG amp Max for the first condition, and Squat sEMG amp Const, Kneeup sEMG amp Const, and Lunge sEMG amp Const for the second.

The fifth metric, iEMG (integrated EMG), calculates the difference in iEMG between the initial and final 10 s of exercise. This metric reflects total muscle activation over time (Phinyomark et al., 2014). It is labeled similarly to the fourth metric and can be obtained only from sEMG signals.

### 2.5 Statistical analysis

For the statistical analysis, we first performed the Shapiro-Wilk test on the muscle parameter data and performance metrics. The results showed *p*-values greater than 0.05 for all muscle parameters, confirming normal distribution, as seen in Tables 2, 3. We then calculated Cohen’s $f^{2}$ value (Selya et al., 2012) and conducted a power analysis to ensure the sample size was adequate (Myors et al., 2010).

We also tested the normality of each performance metric, used as independent variables in the multivariable regression analysis (Tabachnick et al., 2013), using the Shapiro-Wilk test. Additionally, we checked the normality of residuals and homoscedasticity to verify that the selected independent variables met the assumptions required to explain the dependent variable, which was the muscle parameter.

Next, we examined the correlation between muscle parameters and performance metrics using Pearson’s correlation coefficient. To ensure the independence of the performance metrics, we analyzed inter-variable correlations, interpreting the strength of these correlations as negligible ( < 0.1), weak (0.1–0.39), moderate (0.4–0.69), strong (0.7–0.79), and very strong ( > 0.8) (Schober et al., 2018).

These analyses were crucial in identifying the key factors influencing muscle strength. Variables that did not meet the selection criteria were excluded from the final regression model. All statistical analyses were conducted using SPSS version 26.0 (IBM, Armonk, NY) and MATLAB R2020a (MathWorks, Natick, MA, United States).

## 3 Results

### 3.1 Association with muscle parameters and performance metrics

**FIGURE 4:**
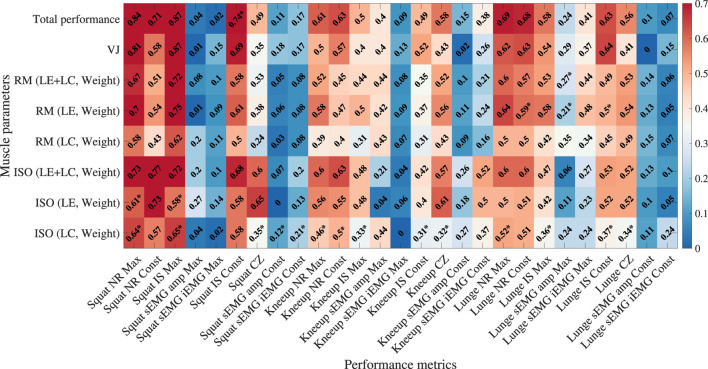
Absolute values of Pearson’s correlation coefficients between performance metrics and muscle parameters were analyzed to assess the linearity of the correlations. Asterisks (*) indicate *p*-values less than 0.05, signifying that the assumption of normality was violated in the residuals’ Shapiro-Wilk test. Red indicates a stronger correlation, while blue represents a weaker correlation.

**FIGURE 5:**
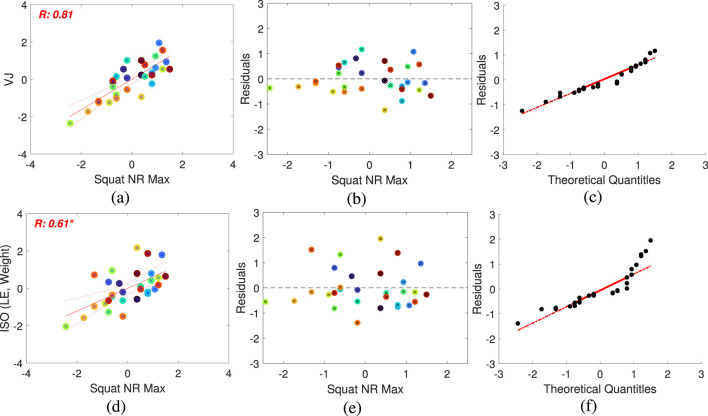
Results of linear regression analysis. **(A)** and **(D)**: Scatter plots showing the relationship between dependent variables (**(A)** for VJ, **(D)** for ISO (LE, Weight)) and the independent variable (Squat NR Max). Pearson’s correlation coefficient values are indicated in red. **(B)** and **(E)**: Residual plots displaying the differences between the model and the independent variable to assess heteroscedasticity. **(C)** and **(F)**: QQ plots comparing the model with the independent variable, assessing heteroscedasticity and normality.

As shown in Figure 4, there is a clear linear association between muscle parameters (Table 2), and certain performance metrics used to evaluate exercise performance with Bot Fit. Brighter colors in the figure indicate a stronger correlation with muscle parameters. However, it is important to note that a high linear correlation alone does not guarantee that a variable is suitable as an independent predictor in the regression model. To ensure the robustness of the model, we performed tests for homoscedasticity and normality of residuals, as depicted in Figure 5.

Among the common variables across the three types of exercises, we observed a strong correlation between the number-based performance metric NR and the initial exercise speed IS. Additionally, among the different types of exercises, squats consistently showed stronger correlations with muscle parameters compared to the other exercises. The scatter plots in Figures 5A, D illustrate the correlations between these variables, each exhibiting a high degree of linearity, with correlation coefficients of 0.5 or higher. However, the residuals reveal varying levels of heteroskedasticity and normality across the models. In Figure 5B, the residuals are evenly distributed, indicating homoscedasticity. In contrast, the models in Figure 5E display unevenly distributed residuals, suggesting the presence of heteroskedasticity. Similarly, while the residuals in Figure 5C follow a normal distribution, those in Figure 5F deviate from normality, as confirmed by the QQ-plots that visualize normality and heteroskedasticity.

Through these analyses, we identified that specific performance metrics, particularly those related to initial exercise speed, serve as statistically significant predictors of muscular strength. When validated through appropriate statistical checks, as outlined in Table 3, these metrics can be reliably used in regression models to predict muscle parameters.

### 3.2 Multivariable analysis

#### 3.2.1 Association between multivariable

**FIGURE 6:**
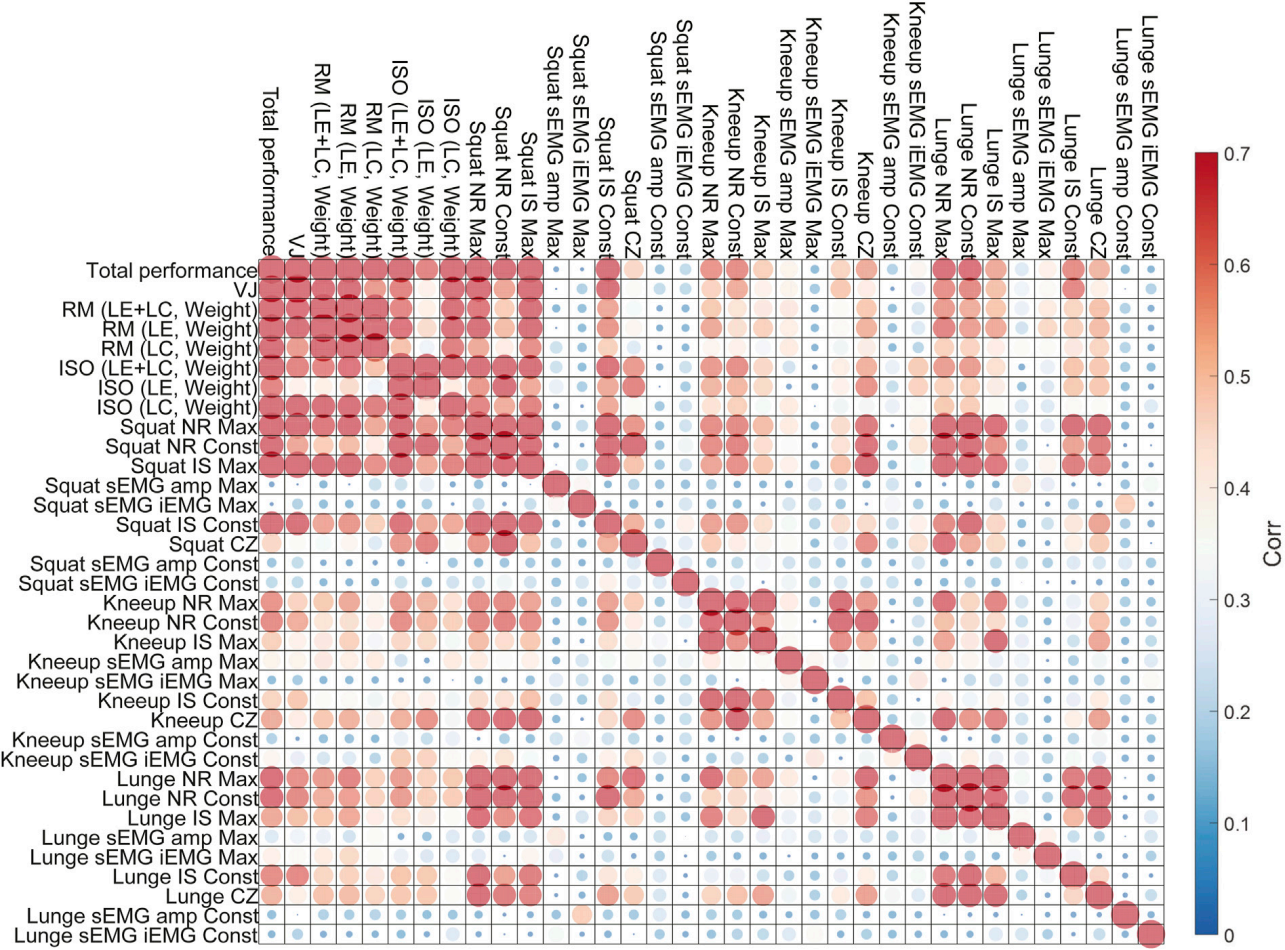
Absolute correlation matrix with muscle parameters and performance metrics is presented in Tables 2, 3. The larger the size and the redder the color of the circle, the stronger the correlation, while the smaller the size and the bluer the color, the weaker the correlation.

As illustrated in Figure 6, the correlation matrix provides critical insights into the relationships among all variables, including both the independent performance metrics and the dependent muscle parameters. This matrix not only highlights how various performance metrics are associated with muscle parameters but also reveals the interrelationships among the performance metrics themselves. This dual insight is essential for refining our understanding of how different aspects of exercise performance, measured through Bot Fit, relate to overall muscle strength.

By analyzing these correlation patterns, we can identify clusters of performance metrics that exhibit strong correlations. For example, certain metrics consistently show high correlations with muscle parameters, suggesting they play a central role in predicting muscle strength. Conversely, metrics that are highly correlated with each other might indicate redundancy, implying that not all need to be included in our regression model. Identifying such clusters helps narrow down the set of independent variables, leading to a more streamlined and efficient model.

This refined selection process is not merely a technical optimization—it has practical implications. By focusing on fewer, yet highly representative metrics, we reduce complexity while maintaining the model’s predictive power. This allows us to make more accurate assessments of muscle strength using Bot Fit without overcomplicating the model with redundant variables.

### 3.3 Clustering between multivariable

**FIGURE 7:**
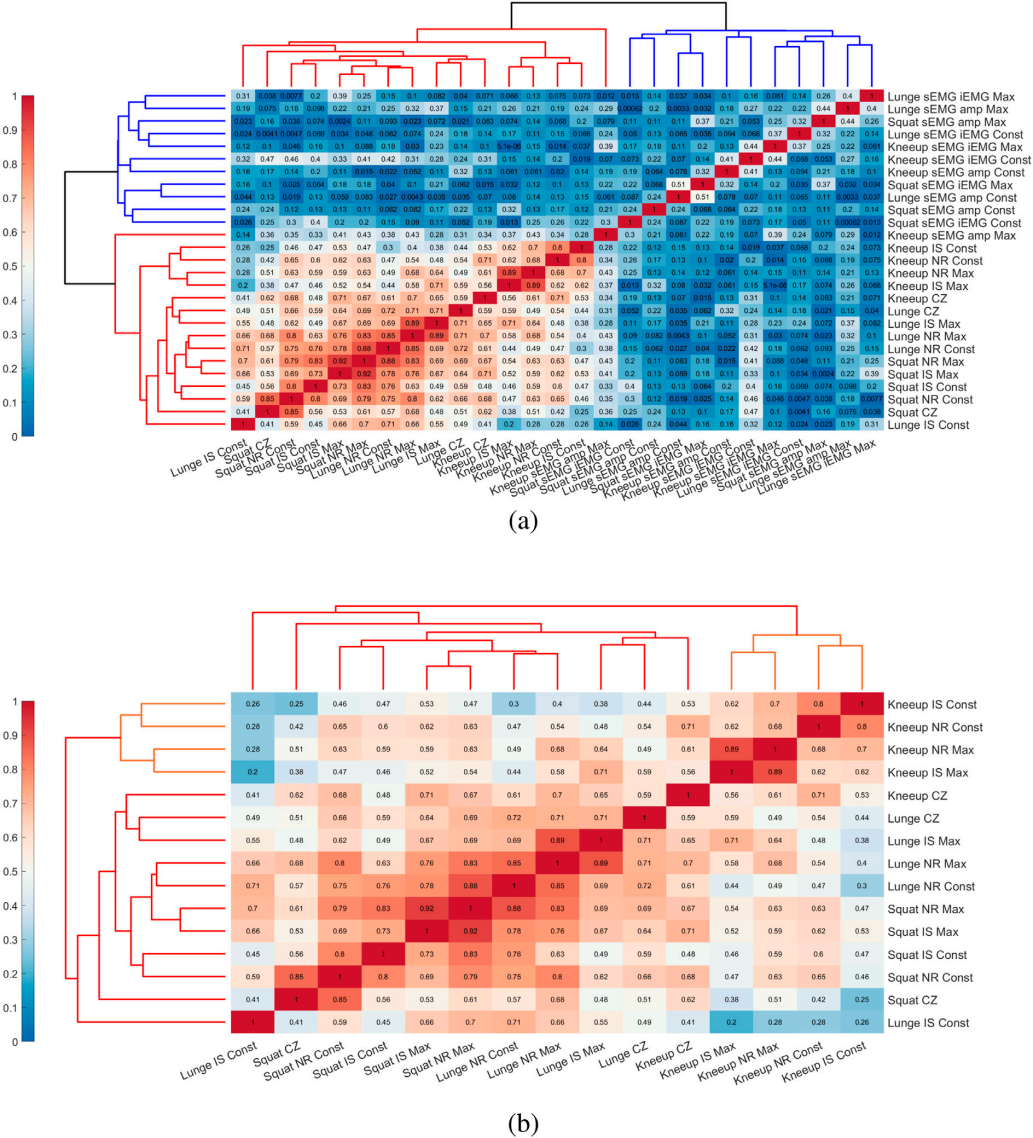
Correlation matrix with dendrogram among the independent variables of performance metrics in Table 3. **(A)** Correlation matrix of all performance metrics. **(B)** Correlation matrix clustered by highly correlated variables from **(A)**. Red indicates a stronger correlation, while blue indicates a weaker correlation.

In the multivariable analysis, we confirmed associations among independent variables and performance metrics, as demonstrated in Figure 6. Through hierarchical clustering in Figure 7, we performed the process of selecting variables with high correlations, indicating lower independence as independent variables. This allows for the simplification of the predictive model. In Figure 7A, variables with high correlations were clustered together, and in Figure 7B, the clustering was made clearer.

In particular, through this dendrogram analysis, we observed that the IS, NR, and CZ metrics related to muscle parameters had a high correlation with Squat, Knee-up, and Lunge, and that these metrics also exhibited a high correlation among themselves. Additionally, Squat and Lunge metrics were classified within the same cluster, while Knee-up was classified independently. This suggests that the metrics for Squat and Lunge could sufficiently represent each other.

**FIGURE 8:**
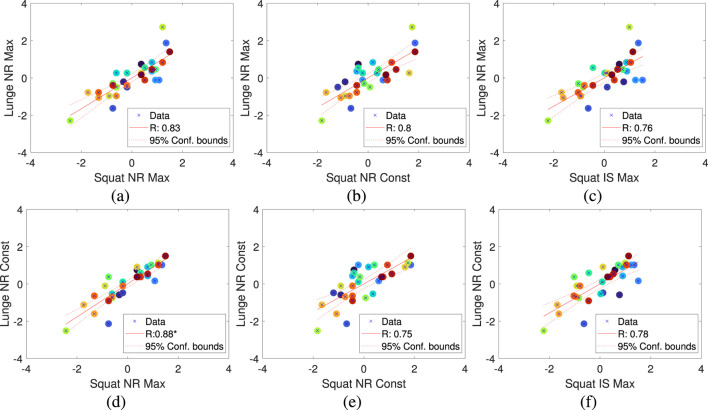
Linear correlation between performance metrics for squat and lunge exercises. **(A)** Correlation between Squat NR Max and Lunge NR Max. **(B)** Correlation between Squat NR Const and Lunge NR Max. **(C)** Correlation between Squat IS Max and Lunge NR Max. **(D)** Correlation between Squat NR Max and Lunge NR Const. **(E)** Correlation between Squat NR Const and Lunge NR Const. **(F)** Correlation between Squat IS Max and Lunge NR Const.

Based on this, we identified meaningful relationships between variables, particularly the strong correlation between Squat and Lunge, as shown in Figure 8. Among the performance metrics, we confirmed that NR and IS showed high correlations between these two exercises.

Specifically, we identified linear relationships between Squat NR Max and Lunge NR Max (Figure 8A), Squat NR Const and Lunge NR Max (Figure 8B), Squat IS Max and Lunge NR Max (Figure 8C), and similarly for Lunge NR Const and Squat NR Max (Figure 8D), Squat NR Const (Figure 8E), and Squat IS (Figure 8F).

**FIGURE 9:**
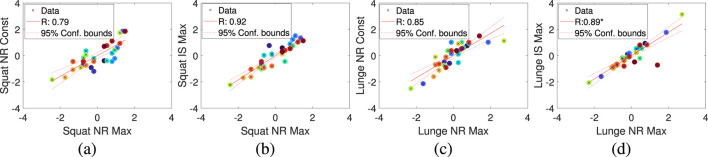
Linear correlation between performance metrics related squat. **(A)** Correlation with Squat NR Max and Squat NR Const. **(B)** Correlation with Squat NR Max and Squat IS Max. **(C)** Correlation with Lunge NR Max and Lunge NR Const. **(D)** Correlation with Lunge NR Max and Lunge IS Max.

Additionally, we compared the performance metrics within Squat and Lunge in Figure 9. Figure 9A illustrates the relationship between Squat NR Max and Squat NR Const, while Figure 9B shows the relationship between Squat NR Max and Squat IS Max. This confirms that the metrics in the Squat protocol are correlated with each other, indicating that one of these metrics could be selected to explain the dependent variable in the regression model, rather than using all of them. Similarly, within the Lunge protocol, Figure 9C represents the relationship between Lunge NR Max and Lunge NR Const, and Figure 9D shows the relationship between Lunge NR Max and Lunge IS Max. As with the Squat protocol, it is also possible to select one metric within the Lunge protocol to explain the dependent variable, as confirmed by the results.The hierarchical clustering of correlated variables, such as Squat and Lunge, allows us to streamline the regression model by selecting only the most relevant metrics. This reduces complexity without compromising accuracy. Strong correlations between NR and IS metrics in both Squat and Lunge indicate that these metrics are strong predictors of muscle performance. Therefore, selecting one representative metric from correlated groups prevents multicollinearity and enhances the model’s robustness. Ultimately, this method optimizes the model, improving both efficiency and interpretability, making it more applicable for systems like Bot Fit.

### 3.4 Regression analysis

#### 3.4.1 Multivariable model

To develop the final multivariable model, we selected the most relevant indicators based on prior statistical analyses. The chosen independent variables are listed in Table 4, which were derived from the performance metrics in Table 3. These variables were selected based on normality (*p*-value > 0.005) (Tabachnick et al., 2013), homoscedasticity, and normality of residuals (Figures 4, 5). To minimize multicollinearity, only variables with linearity below 0.7 (Murray and Conner, 2009) were included (Figures 7–9).

**TABLE 4:** Selection of independent variables (performance metrics) for a regression model targeting muscle parameters through statistical validation.

	Model target; muscle parameters			
	Total performance	VJ	RM (LE + LC, Weight)	ISO (LE + LC, Weight)
Independent variables Selected performance metrics (Criteria: normality test; *p* > 0.05, residual normality and homoscedasticity test, independence test; linearity: < 0.7)	Squat IS Max	Squat IS Max	Squat IS Max	Squat IS Max
	Kneeup IS Max	Kneeup NR Max	Kneeup CZ	Kneeup CZ
	Kneeup CZ	Lunge IS Max	Lunge IS Max	Lunge CZ
	Lunge IS Const		Lunge CZ	
	Lunge CZ			

**FIGURE 10:**
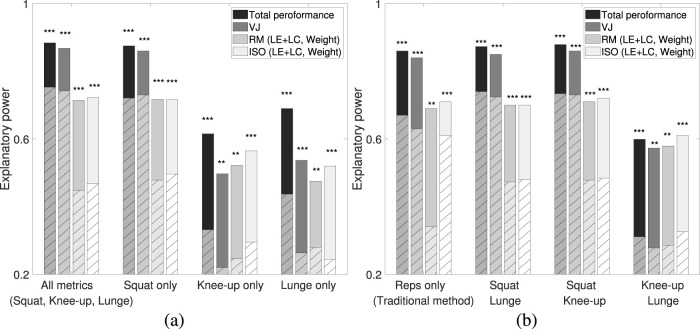
Performance of each regression model for Total performance, VJ, RM (LE + LC, Weight), and ISO (LE + LC, Weight). Solid bars represent the model’s explanatory power (R), while hatched bars represent the adjusted R^2^. **(A)** “All metrics” includes metrics from Squat, Knee-up, and Lunge; “Squat only” uses squat-related metrics; “Knee-up only” uses knee-up-related metrics; and “Lunge only” uses lunge-related metrics. **(B)** “Reps only” represents a traditional strength assessment method using metrics related to the number of repetitions (NR) (Schoenfeld et al., 2019). “Squat & Lunge,” “Squat & Knee-up,” and “Knee-up & Lunge” include metrics from the respective combinations of exercises. This figure shows model performance based on high correlations and statistically significant metrics for each target, categorized by resistance exercise type. Asterisks indicate significance levels: * *p* < .05, ** *p* < .01, and *** *p* < .005.

The model’s performance is illustrated in Figure 10. In Figure 10A, the explanatory power for each target is shown using metrics from all three exercises (All metrics) and from individual exercises (Squat-only, Knee-up-only, and Lunge-only). The results indicate that using all three exercise metrics produced similar performance to using Squat metrics alone, whereas models based solely on Knee-up or Lunge metrics had lower performance.

Figure 10B compares the model’s performance across different exercise combinations. Models incorporating Squat metrics consistently performed the best, with the Squat and Knee-up combination yielding the highest accuracy. This confirms that Squat-related metrics are the strongest predictors of muscle strength. Interestingly, the combination of Squat and Lunge metrics performed similarly to the Squat-only model, likely due to the similarities in movement patterns between these exercises. Conversely, Knee-up metrics were more distinct, and combining Squat and Knee-up metrics resulted in more efficient muscle strength predictions than using all three exercises together.

Additionally, we compared our models to a traditional method (Schoenfeld et al., 2019) that assesses strength based on repetition counts (NR) from the three exercises. Our proposed models—whether using Squat alone or combined with Knee-up—performed slightly better than the traditional approach, as shown in Figure 10B. This demonstrates that our method can accurately estimate muscular strength without the need for all exercises to be performed.

Based on these results, we finalized the multivariable regression models, presented in Table 5. The “All metrics” model includes data from all three exercises, the “Squat, Knee-up” model uses only two exercises, and the “Squat-only” model relies solely on Squat metrics. Notably, the performance of the “Squat-only” model was comparable to that of the “All metrics” model, as shown in Figure 10.

**TABLE 5:** Multiple linear regression models for total performance with squat, knee-up and reverse lunge. Beta coefficients (B) signify the estimated change in the dependent variable for a one-unit change in the predictor variable, holding all other variables constant. Standard errors (SE) gauge the precision of the estimated coefficients. Standardized coefficients (Standardised coeff. Beta) indicate the change in standard deviations of the dependent variable for a one-standard-deviation change in the predictor variable. Cohen’s $f^{2}$ (Selya et al., 2012) is a measure of effect size in regression models, used to assess how much an independent variable explains the variance of the dependent variable. Statistical power (Myors et al., 2010) refers to the probability that the test correctly rejects the null hypothesis when a true effect exists, with a value of 1.0 indicating that the sample size and effect size are sufficient to detect a statistically significant result.

Type of independent variables	Independent variables	Unstandardised coeff		Standardised coeff	R (adjust $R^{2}$ )	Effect size; Cohens’s $f^{2}$ (Statistical power)
		B	SE	Beta		
All metrics (Squat, Knee-up, Lunge)	(Constant)	−5.203	1.374	—	0.884 (0.753***)	Large; $f^{2}$ = 3.57 Power ≈ 1.0
	Squat IS Max	0.626	0.131	0.829		
	Kneeup IS Max	0.046	0.042	0.142		
	Kneeup CZ	−0.001	0.002	−0.111		
	Lunge IS Const	−0.022	0.023	−0.128		
	Lunge CZ	−0.003	0.007	−0.057		
Squat Knee-up	(Constant)	−5.803	0.706		0.879 (0.734***)	Large; $f^{2}$ = 3.26 Power ≈ 1.0
	Squat IS Max	0.632	0.082	0.837		
	Kneeup IS Max	0.022	0.036	0.068		
Squat only	(Constant)	−5.58	0.602		0.873 (0.721***)	Large; $f^{2}$ = 3.2 Power ≈ 1.0
	Squat IS Max	0.659	0.07	0.873		

** *p* < 0.01; *** *p* < 0.005. $f^{2}$ : < 0.02 (small effect), $f^{2}$ : 0.02–0.15 (medium effect), and $f^{2}\;>$ 0.35 (large effect).

To determine the appropriate sample size for the final model, we conducted a statistical power analysis, including *p*-values and effect sizes, ensuring that the model is reliable and can generalize effectively.

Among the various models, the “Squat-only” model using the Squat IS MAX metric, which measures speed over a 10-s interval, showed the best performance. This confirms that Squat IS MAX is a highly reliable and efficient predictor of muscle strength. Statistical analysis further validated the model’s applicability, making it suitable for scenarios with limited resources or smaller sample sizes.

#### 3.4.2 Multivariable model validation

To assess the impact of sample size, age, and gender on the model, we employed a bootstrapping technique (Wang, 2019). Additionally, we introduced Gaussian noise into the bootstrapped datasets (Gu et al., 2019) to evaluate the influence of factors such as gender, age, and participant condition. As outlined in Table 6, bootstrapping was performed using data from the original 30 participants, generating 100 and 300 samples, with 10% and 30% Gaussian noise added to each model. We then evaluated the performance of each regression model.

**TABLE 6:** Regression model performance. The dependent variables of each regression were determined by fixing the mean and standard deviation of the dataset obtained in this study and applying z-score normalization to the other bootstrapped datasets. Accordingly, all results were derived from the independent variables (i.e., All metrics, Squat and Knee-up, Squat only as shown in Table 5) with the dependent variable set as ‘Total Performance’. The explanatory power of the model, represented by the R value (adjusted $R^{2}$ ), is also shown. Additionally, a comparison of results with and without gender and age included as independent variables is provided. For gender, we applied a value of male:1/female:0 as an independent variable.

Type of independent variables	Gender and age not included					Gender and age included				
	30 samples	Bootstrapped 100 samples		Bootstrapped 300 samples		30 samples	Bootstrapped 100 samples		Bootstrapped 300 samples	
	Original	Noise 10%	Noise 30%	Noise 10%	Noise 30%	Original	Noise 10%	Noise 30%	Noise 10%	Noise 30%
All metrics (Squat, Knee-up, Lunge)	0.884 (0.753***)	0.854 (0.733***)	0.814 (0.718***)	0.833 (0.718***)	0.803 (0.615***)	0.891 (0.741***)	0.857 (0.733***)	0.817 (0.719***)	0.841 (0.721***)	0.806 (0.616***)
Squat Knee-up	0.879 (0.734***)	0.845 (0.708***)	0.81 (0.700***)	0.819 (0.695***)	0.79 (0.596***)	0.888 (0.746***)	0.851 (0.713***)	0.817 (0.707***)	0.834 (0.71***)	0.796 (0.612***)
Squat only	0.873 (0.721***)	0.84 (0.711***)	0.81 (0.702***)	0.815 (0.691***)	0.780 (0.592***)	0.88 (0.738***)	0.848 (0.716***)	0.815 (0.707***)	0.826 (0.697***)	0.786 (0.604***)

** *p* < 0.01; *** *p* < 0.005.

To compute the total performance of the bootstrapped data, the mean and standard deviation of the muscle parameter from the original 30 participants were used as the model target. This methodology allowed us to evaluate the generalizability of the model to the broader population and assess the sample’s representativeness.

As presented in Table 6, model performance decreased slightly as the number of bootstrapped samples increased, with further reductions observed when higher levels of noise were introduced. However, despite these declines, the changes were not statistically significant, suggesting that the proposed model remains robust and applicable across various data distributions. The effects of gender and age were also apparent, but they did not significantly impact the overall model performance.

Our analysis demonstrates that incorporating all three exercise types (Squat, Knee-up, and Lunge) yields a highly accurate prediction model for muscle strength estimation, as shown in Table 5. However, this approach requires performing all exercises, which may not always be practical. To develop a more efficient model, we identified Squat metrics as the strongest independent predictors of muscle strength. Combining Squat and Knee-up metrics offered the highest explanatory power, while using Squat metrics alone provided a simpler yet highly effective alternative.

The validation results using bootstrapping confirm that the model is robust and generalizable, as shown in Table 6. Although the introduction of noise and an increase in sample size slightly reduced performance, the declines were not statistically significant. This indicates that our final model, particularly the “Squat-only” and “Squat, Knee-up” configurations, can be applied effectively across diverse data sets and conditions. Therefore, the model not only demonstrates strong predictive accuracy but also maintains reliability in scenarios with varied sample sizes and external factors such as age and gender.

## 4 Discussion

Our study explored methods for estimating users’ physical strength through exercises provided by Bot Fit. We found that strength could be effectively estimated using statistically significant prediction models derived from performance metrics in resistance exercises such as squats, knee-ups, and reverse lunges performed with Bot Fit. Notably, even a single exercise—the squat—produced a model with predictive accuracy comparable to models incorporating all three exercises, highlighting Bot Fit’s capability to generate accurate and efficient strength estimation models based on user performance.

Assessing muscle strength is critical for health management and exercise planning, as it helps individuals understand their capabilities and address deficits. Muscle strength is also recognized as a clinical indicator of overall health status (Momma et al., 2022). Our study successfully developed statistically robust models through resistance exercises facilitated by the Bot Fit system, suggesting that Bot Fit not only supports exercise but also serves as a valuable tool in health monitoring and fitness optimization.

Traditional methods of measuring muscle strength through resistance include the N-RM method, which estimates the 1RM by assessing the maximum weight a person can lift in a single repetition (Willardson and Bressel, 2004), and isokinetic dynamometry, which evaluates strength by maintaining a constant movement speed using specialized equipment (Gleeson and Mercer, 1996; Schoenfeld et al., 2019). Functional strength tests using resistance exercises, like squats, offer alternatives without specialized equipment by analyzing performance metrics such as oxygen consumption and aerobic energy expenditure (Fujita et al., 2016; Nakagata et al., 2022). However, these approaches often require specific equipment, designated exercise locations, and supervision, and they lack the ability to monitor real-time performance data.

Exoskeleton robots offer a promising solution to these limitations. They enable users to perform resistance exercises without the need for specific equipment or locations, and their motor data can be used to monitor movements. While previous studies have focused on using exoskeletons to assist movement and guide exercises (Lee et al., 2023; Kim et al., 2023), tracking users’ gait trajectories and providing walking adaptability for the robot (Kou et al., 2024), research on utilizing exoskeletons to estimate strength in healthy individuals remains limited.

We introduced Bot Fit, an exoskeleton designed to assess users’ functionality through safe and adaptive resistance exercises (Lim et al., 2019; Kim et al., 2018). Previous research has used electromyographic fatigue threshold (EMGFT), biomechanical assessments, and subjective ratings to evaluate muscle strength and ankle-joint stability (Byeon et al., 2024). Technologies like lower-limb wearable robots (LEEX) have also been developed to analyze gait based on biomechanical patterns and movement intentions (Qiu et al., 2023). Our study demonstrated the feasibility of estimating muscle strength in healthy individuals using Bot Fit, offering more challenging resistance exercises compared to bodyweight exercises.

We designed an exercise protocol using Bot Fit under two conditions. The first simulates estimating 1RM through repeated n-RM sets without a fixed speed over a short period, allowing participants to exert maximum effort (Willardson and Bressel, 2004). The second focuses on endurance by maintaining a constant speed over a longer period, encouraging sustained use of strength and endurance. Research shows that faster repetition speeds lead to greater strength gains (Westcott et al., 2001), and speed endurance training improves overall performance (Iaia et al., 2015). Based on the relationship between strength and endurance (Vaara et al., 2012), we used this protocol to estimate individuals’ overall muscular strength.

Results suggest that Bot Fit can efficiently estimate lower body strength, with squats and knee-ups being the most effective exercises. In protocols requiring rapid movement, initial speed-based performance metrics were statistically significant predictors in the strength estimation model. The importance of early speed in representing strength aligns with research highlighting that overcoming inertia to generate fast, powerful movements is closely related to muscle hypertrophy and muscle length (Wilk et al., 2021).

To obtain these performance metrics, we used real-time motor signals from Bot Fit supplemented with data from sEMG sensors, accurately assessing the relationship between initial movement speed and muscle strength. We demonstrated that movement-based metrics such as IS, NR, and CZ can be derived from both sEMG signals and Bot Fit’s motor signals, yielding identical results. This confirms the reliability of Bot Fit’s motor signals in representing actual muscle movement. Simplifying sEMG signal processing by using peak values allowed more efficient extraction of these key metrics.

sEMG signal analysis has significant advantages for evaluating muscle movement and activity. It has been applied to systems predicting wrist joint strength based on musculoskeletal models (Zhang et al., 2016), and methods for estimating lower limb strength using machine learning techniques with sEMG signals have been developed (Mokri et al., 2022). Additionally, EMG-based musculoskeletal models have been used to predict joint moments under various dynamic contraction conditions (Lloyd and Besier, 2003).

However, these studies require complex data processing and parameter calibration, increasing overall complexity (Zhang et al., 2016; Buchanan et al., 2004). Machine learning-based methods, while achieving high prediction accuracy, require large datasets (Mokri et al., 2022). They often use limited parameters and may not account for physiological phenomena like nonlinear changes in muscle strength, reducing predictive power (Lloyd and Besier, 2003). In contrast, we found that amplitude and iEMG values from sEMG showed lower correlation with muscle strength, supporting previous research indicating a nonlinear relationship between sEMG signals and muscle strength (Sartori et al., 2012; Buchanan et al., 2004).

Our findings indicate that a simple approach based on peak movement signals, rather than complex musculoskeletal models (Jiang et al., 2020), suffices for calculating IS, NR, and CZ. These movement-based metrics strongly correlate with muscle strength and are key variables in strength estimation models, aligning with prior studies that use movement speed and repetition counts as indicators of strength (Nakagata et al., 2022).

The regression model developed demonstrates that Bot Fit can effectively estimate lower body strength. Based on resistance exercises like squats, knee-ups, and reverse lunges, the model provided reliable strength predictions. Notably, using data from the squat exercise alone achieved prediction accuracy comparable to models using multiple exercises, highlighting Bot Fit’s potential as a simple and efficient tool for assessing strength.

Our model aligns with previous research exploring the correlation between strength and exercise performance. For example, Rodrigues et al. found a strong relationship between lower body strength, measured by a sit-to-stand test, and dynamic balance assessed via the Timed-Up and Go test (Rodrigues et al., 2023). Similarly, Monteiro et al. developed strength estimation models based on walking parameters, comparable to the knee-up exercise in our study (Monteiro et al., 2021).

Moreover, our model relates to widely used strength assessments like vertical jump performance (Carlock et al., 2004), 1RM tests for leg extensions and curls (Grgic et al., 2020; Kanada et al., 2018), and isometric tests (Bandinelli et al., 1999). Unlike traditional methods that do not incorporate real-time performance data from wearable exoskeletons, our study introduces a more comprehensive performance metric by combining motor signals and sEMG data from the Bot Fit exoskeleton. Real-time signals such as initial exercise speed and the number of repetitions provide a dynamic and precise understanding of muscular strength during resistance exercises. We also compared a model based solely on repetition count with our multivariable model, confirming that our model had superior explanatory power. This further validates our regression model as an effective tool for strength evaluation and exercise planning using Bot Fit.

The multivariable analysis in our study goes beyond simple correlation or regression commonly employed in previous research. By systematically considering multicollinearity and avoiding redundancy, we selected the most critical performance metrics (e.g., initial speed during squats), ensuring that the final model efficiently and accurately predicts lower limb strength across various exercises.

Our study has several limitations. First, the sample population was limited to healthy adults aged 23 to 30, which restricts the generalizability of the model to broader populations, such as older adults or individuals with musculoskeletal conditions. While this limitation narrows the scope of application, the high statistical power of our data indicates that our methods and findings are robust for this specific demographic. Additionally, based on the consistent results across diverse data distributions using our proposed analysis method, we have confirmed the potential generalizability of our approach.

Second, although we aimed to minimize the influence of external variables, we could not fully control the participants’ daily activity levels outside the experimental sessions. While daily physical activity may have had some influence on the results, we believe its impact was minimal. We monitored participants’ baseline heart rates and conducted preliminary exercises to ensure consistent conditions at the start of each session.

Lastly, the study’s focus on using a wearable exoskeleton for fitness movements primarily applies to physically active individuals. Nevertheless, our results suggest that Bot Fit has potential beyond fitness assessments. The integration of exercise speed and performance metrics into our model introduces a novel approach to strength evaluation, highlighting the versatility of exoskeletons for broader fitness and health applications.

## 5 Conclusion

This study demonstrates the practicality and effectiveness of using Bot Fit to estimate lower body strength through key performance metrics such as IS, NR, and CZ. Among these, IS emerged as the most accurate predictor, reflecting the user’s ability to overcome inertia and perform quick, forceful movements. By focusing on simple resistance exercises like squats and knee-ups, we developed strength prediction models that are both reliable and easy to implement. Notably, strength estimates based on a single exercise, such as the squat, were comparable to those derived from multiple exercises, highlighting Bot Fit’s ability to provide streamlined and efficient strength assessments. These metrics were carefully selected through rigorous statistical analysis, resulting in a robust and accurate estimation model.

Our study introduces a novel approach compared to traditional methods of predicting lower limb strength, demonstrating Bot Fit’s potential as a valuable tool beyond rehabilitation. By incorporating sEMG-based evaluation metrics and performance data from Bot Fit’s motor signals, we used multivariable analysis to identify the most effective predictors—such as initial exercise speed and the number of repetitions—allowing for more precise assessments of muscle strength. The resulting strength prediction model, particularly when using squat metrics, showed significantly higher accuracy than conventional methods, underscoring the potential for exoskeleton robots to be applied in real-world fitness and health monitoring systems. Bot Fit offers a simple and reliable method for tracking strength in healthy individuals, without the need for specialized equipment or environments.

Future study should include older adults and individuals with musculoskeletal conditions to validate the model’s generalizability. Exploring additional exercise protocols could help develop models suited to diverse physical abilities and rehabilitation needs. Leveraging advanced data analytics and machine learning could further enhance strength prediction accuracy, enabling Bot Fit to deliver personalized exercise recommendations and health assessments to a wider range of users.

## Data Availability

The data files are available from the corresponding author upon reasonable request.
